# An exploratory study of psychosis risk factors in individuals who are referred but do not meet criteria for an early intervention in psychosis service

**DOI:** 10.1192/bjo.2023.640

**Published:** 2024-01-05

**Authors:** Sean Naughton, Aoife Brady, Eoin Geary, Eimear Counihan, Mary Clarke

**Affiliations:** Dublin and East Treatment and Early Care Team (DETECT) Early Intervention in Psychosis Service, Dublin, Ireland; and School of Medicine, University College Dublin, Ireland; Dublin and East Treatment and Early Care Team (DETECT) Early Intervention in Psychosis Service, Dublin, Ireland

**Keywords:** Early intervention in psychosis, at-risk mental state, youth mental health, first-episode psychosis, clinical high risk

## Abstract

**Background:**

The ‘at-risk mental state’ (ARMS) for psychosis has been critiqued for its limited prognostic ability and identification of a limited proportion of those who will develop a first episode of psychosis (FEP). Broadening the search for high-risk groups is key to improving population-level ascertainment of psychosis risk.

**Aims:**

To explore risk enrichment in diagnostic, demographic and socio-functional domains among individuals referred to an early intervention in psychosis (EIP) service not meeting ARMS or FEP criteria.

**Method:**

A retrospective file review of 16 years of referrals to a tertiary EIP service in Ireland was undertaken. Diagnostic outcomes from standardised assessments (Structured Clinical Interview for DSM), demographic (age, gender, family history, nationality) and socio-occupational (relationship status, living status, working status) variables were compiled for those not meeting criteria. These were compared with individuals diagnosed with an FEP in the same period.

**Results:**

From 2005 to 2021 inclusive, of 2025 index assessments, 27.6% (*n* = 558) did not meet either FEP or ARMS criteria, which is notably higher than the 5.4% (*n* = 110) meeting ARMS criteria. This group had high psychiatric morbidity, with 65.4% meeting criteria for at least one DSM Axis I disorder. Depressive, anxiety and substance use disorders predominated. Their functional markers were poor, and comparable to the FEP cohort.

**Conclusions:**

This group is enriched for psychosis risk factors. They are a larger group than those meeting ARMS criteria, a finding that may reflect EIP service configuration. They may be an important focus for further study in the search for at-risk populations beyond the current ARMS model.

## The limitations of positive symptom spectrum ‘at-risk mental state’ models

Early intervention in psychosis (EIP) services have become well-embedded in mental health services over the past two decades, with a focus on the early detection and treatment of first-episode psychosis (FEP).^1^ The paradigm further developed to primary and secondary prevention, identifying individuals with an at-risk mental state (ARMS) of developing psychosis. These criteria focus primarily on the detection of sub-disorder threshold positive psychotic symptoms.^2^ This ARMS model has a modest predictive value for psychosis in selected samples,^3^ and recent critiques have highlighted this limited predictive ability, the uncertain mutability of transitions rates to psychosis at this stage, and the identification of only a narrow group of individuals at risk of a psychotic disorder.^4,5^

Despite calls to broaden the ARMS paradigm,^6^ this has not resulted in any revision of current criteria. In contrast, other predictive models of medical risk have developed around a system of iterative enhancements. The QRISK model for cardiovascular disease risk has had annual updates since 2007, adding and reweighting risk factors on the basis of population data. Currently in its third major revision, this development has resulted in increased ascertainment of cardiovascular events.^7^ These systems are designed for the identification of high-risk groups within the general population, and are therefore built around known risk factors for the condition rather that identifying individuals with subthreshold symptoms of the target pathology.

Although several enhanced prediction models are under evaluation, the majority are derivative of ARMS criteria, combining biological and clinical markers in this group.^8^ Although this may increase the specificity of prediction, the sensitivity will remain challenged. The attenuated positive symptoms that characterise the ARMS state may not be a universal, or even common, entry point to psychosis. In locations where ARMS services are well established, only a minority with incident psychosis have initially had this diagnosis.^9,10^ Population-based studies put ARMS sensitivity for later psychosis as low as 15%.^11^ This has led to calls for a broader starting point to defining high-risk groups.^12^

## The search for at risk-groups beyond the current ARMS model

Because of the relatively low incidence of psychotic disorders in the general population, defining high-risk groups remains essential for any workable prediction model.^13^ Recent work to identify such groups have had success utilising ‘clinical pathway risks’. Attendance at child and adolescent mental health services and presentation to an emergency department following self-harm as an adolescent, which do not incorporate any specific test for psychosis spectrum symptoms, have demonstrated predictive accuracy for psychosis superior to many ARMS samples.^14,15^ This may be reflective of a common set of risk factors for psychosis shared by individuals referred for assessment or treatment of any significant psychiatric symptoms. Indeed, individuals referred for assessment of psychosis and psychosis risk are already selectively filtered through a range of consecutive referral processes,^13^ resulting in a group substantially enriched for known psychosis risk factors, many of which are not components of the current ARMS model.^16^ This sample enrichment likely accounts for a large proportion of the ARMS post-test risk.^17^

Such risk factors include symptomatic and functional markers that are established antecedents of psychotic disorders. Substantial non-psychotic psychiatric morbidity is known to precede the development of psychosis, with anxiety and depressive disorders the plurality.^18,19^ At a general population level, mood disorders have a higher population attributable fraction for psychosis than ARMS status.^20^ Socio-occupational functional impairments are also antecedent markers of psychosis, manifesting in both the premorbid and prodromal phases.^21,22^ Such markers generally appear earlier than positive symptoms,^23^ offering the potential for intervention within a developmental window of sensitivity.^24^

## Rationale for the current study

The current study is an exploratory study of risk factors in individuals referred to a tertiary EIP service because of a concern from a referring clinician about psychosis risk, but without symptoms sufficient for either ARMS or FEP status. Although this is little studied group, the limited research to date indicates they constitute a significant proportion of EIP service referrals, at 41–59%.^25–27^ They have been reported as having high general psychiatric morbidity^28^ and substantial occupational impairment.^27^

We hypothesise that these individuals are enriched for known psychosis risk factors, even in the absence of positive spectrum symptoms sufficient for ARMS or FEP status, and thus may constitute an important group for further study. This study compiled information on demographic, non-psychotic psychiatric morbidity and socio-occupational variables. As a reference for risk enrichment and functioning within this cohort, they are compared with individuals diagnosed with an FEP during the same time period.

## Method

### Study setting

The Dublin and East Treatment and Early Care Team (DETECT) is a tertiary EIP service in Ireland. The service commenced operation in 2005, with initial funding from the St. John of God Hospitaller Services Group, and later the publicly funded Health Service Executive. It is configured as a hub and spoke model.

This public service provides universal access within a geographically defined catchment area in the East Coast Region. The catchment area is defined by community healthcare organisation six (CHO6), with a population of over 400 000. It includes a mixture of urban and rural areas and varying levels of socioeconomic deprivation. CHO6 is served by three adult mental health services: Cluain Mhuire, Dublin South East and East Wicklow. It also includes an independent sector organisation St. John of God Hospital, which provides care on a funded basis. Referrals to DETECT are accepted only from these secondary care sources, and primary care or self-referral is not possible. Referrals are accepted for those aged 18–65 years, in-patients or out-patients, where there are concerns that an individual's presenting symptoms are suggestive of an early-stage psychotic illness.

DETECT provides a comprehensive assessment for all referrals and access to specialist interventions for individuals experiencing an FEP. Those who do not meet the threshold for a psychosis spectrum disorder but do meet criteria for a psychosis risk syndrome are offered reassessment with a standardised instrument at 6 months, to investigate the course of symptoms, or earlier if there are clinical concerns. For those experiencing psychosis, but not in their first episode (‘psychosis, not FEP’), or who do not meet criteria for any of the categories (‘not meeting criteria’), specialist interventions or review are not offered, but a comprehensive diagnostic report is provided to the referring clinical team. Re-referral of those ‘not meeting criteria’ are encouraged if there is any change in their symptoms.

### Assessment and case criteria

Individuals referred to DETECT, who are agreeable to assessment, participate in a comprehensive structured diagnostic interview. From the inception of the service all assessments have included the Structured Clinical Interview for DSM (SCID). Modules A to G are utilised, assessing for mood disorders, psychotic disorders, anxiety disorders, substance use disorders, obsessive–compulsive and related disorders, and their differentials. Outcomes are recorded for all diagnoses met, with the primary disorder assigned as the disorder that is the subject of the referral symptoms. The Structured Interview for Psychosis-Risk Syndromes (SIPS) is administered where there is a clinical concern of a psychosis-risk syndrome (ARMS). All assessments are carried out by experienced clinicians of the EIP service trained in the administration of structured diagnostic assessments.

The case criteria for the service include those experiencing a psychotic illness of any aetiology, including substance-induced psychosis and affective illnesses with secondary psychotic features. There is no exclusion criterion relating to antipsychotic administration. Individuals experiencing psychotic symptoms for more than 2 years do not meet case criteria, but in this study they have been allocated to a separate cohort ‘psychosis, not first episode’ and are not included in the definition of ‘not meeting criteria’. The case criteria have not altered since service inception.

### Data collection

A registry of referrals has been maintained since the service was established. An electronic report is compiled at each assessment, including the outcome of the SCID assessment, and demographic and functional details. The registry was searched for all referrals from the beginning of the service in 2005 to the end of 2021, a 16-year timespan. The diagnostic outcome and relevant details of those who completed an assessment were compiled.

Socio-occupational functional markers included relationship status, living status and current working status. These attributes were collected in a standardised report template, and grouped into categories for the study. Current working status, incorporating either engagement in paid employment or education, was recorded as working or in education, short-term unemployment (≤12 months), long-term unemployment (>12 months) or unable to work (retired, disabled or legally unable to work).

Membership of each cohort was defined by status at initial assessment. Re-referrals to the service were identified and assessments compiled to determine diagnostic change. Assessment of conversion to psychosis was determined only by repeat assessment in DETECT, which depended on an individual being re-referred to the service. Clinical files outside of the EIP service were not examined to determine if conversion to psychosis may have occurred in the absence of a re-referral to the service.

### Study outcomes

The first aim of this study is a descriptive analysis of risk factors, in diagnostic, demographic and functional domains, among individuals referred to, but not meeting criteria for, an EIP service. The second aim is a comparative analysis of demographic and socio-occupational variables with a cohort meeting criteria for an FEP during the same interval.

### Data analysis

Assessment outcomes were grouped by cohort and presented as counts and proportions. Variations in the proportion of each cohort over the study period are present as annual totals and trends. Diagnostic outcomes for the ‘not meeting criteria’ group are presented as totals for each DSM diagnosis and also grouped by category.

Cohorts were compared with *t*-tests for continuous variables, chi-squared tests for binary independent variables and binary logistic regression for multicategory independent variables, and reported as odds ratios. The comparative analysis of functional variables was adjusted for demographic factors (gender, age and nationality) in a binary logistic regression, and reported as adjusted odds ratios. Data were analysed with SPSS software version 28 for Windows.

### Ethical statement and approval

The authors assert that all procedures contributing to this work comply with the ethical standards of the relevant national and institutional committees on human experimentation and with the Helsinki Declaration of 1975, as revised in 2008. This study employed a retrospective file review methodology, and as such individual written consent from participants was not required, consistent with the Irish Data Protection Act 2018 (Section 36(2)) (Health Research) (Amendment) Regulations 2021. All procedures involving human patients, including compliance with the conditions of the 2021 Regulations, were reviewed by the St. John of God Hospitaller Research Ethics Committee and approved under ID797.

## Results

### Outcome of all service referrals and trends

**Fig. 1:**
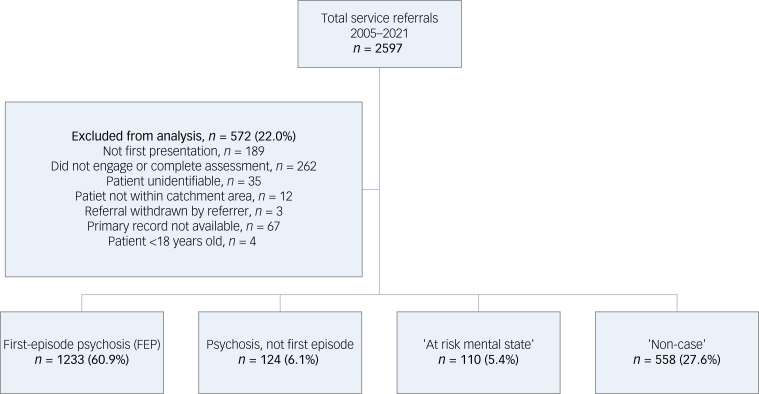
Flow chart of total service referrals.

From the commencement of the service in 2005 to the end of the study period at the end of 2021, there were 2597 referrals received (Fig. 1). Of those, 10.0% (*n* = 262) did not engage with or complete the assessment. A further 7.2% (*n* = 189) were re-referrals, which included both index cases and non-cases. A small number of referrals did not proceed to assessment, either because the patient was not within the EIP service catchment area (*n* = 12) or the referral was subsequently withdrawn (*n* = 3). There were four individuals under 18 years of age. A small proportion of individuals (1.3%, *n* = 35) were recorded only by their initials in the registry, with no other identifying information, so their initial assessments could not be located. Finally, for 2.5% (*n* = 67) of referrals with complete details, a corresponding initial assessment could not be located. This yielded 2025 index referrals who completed a full assessment.

Of all index referrals over the study timeframe, 60.9% (*n* = 1233) met criteria for an FEP. A further 6.1% (*n* = 124) were experiencing a psychotic disorder, but not in their first episode, and 5.4% (*n* = 110) met ARMS criteria. This yielded approximately a quarter (27.6%, *n* = 558) of all referrals who did not meet criteria for either ARMS or FEP status, and had never experienced previous psychotic symptoms.

**Fig. 2:**
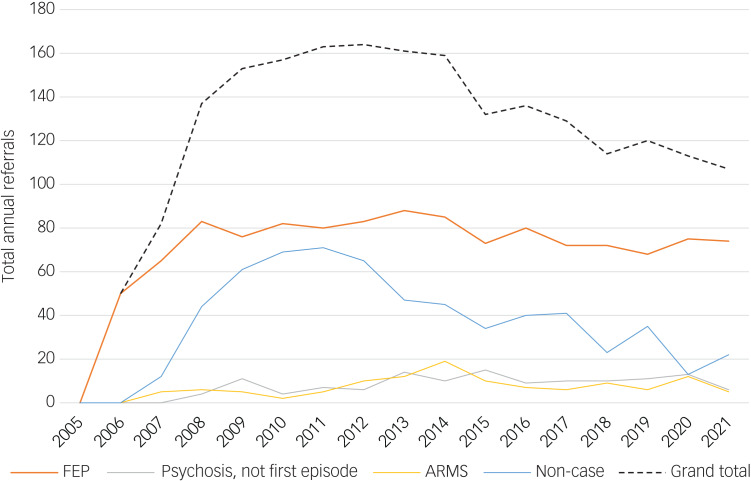
Trend of annual referral count by cohort. ARMS, at-risk mental state; FEP, first-episode psychosis.

The proportion of those ‘not meeting criteria’ varied over the course of the study period, despite the absolute size of other cohorts remaining relatively constant (Fig. 2). This cohort trended to almost match FEPs as a proportion of the overall referral caseload in the first 4 years of operation of the service, thereafter declining steadily.

### Diagnostic profile of those ‘not meeting criteria’

The majority (65.4%, *n* = 366) of individuals in the ‘not meeting criteria’ cohort did meet criteria for at least one DSM Axis I disorder (Table 1). The most common diagnostic groups as primary diagnoses were depressive disorders (22.8%, *n* = 127) followed by anxiety disorders (18.1%, *n* = 101). Substance use disorders were also common as primary diagnoses (14.7%, *n* = 82), with alcohol use disorder representing over half of this group (53.7%, *n* = 44). Finally, bipolar affective disorder accounted for 4.5% (*n* = 25) of the cohort, with type 1 bipolar disorder the predominant diagnosis (72.0%, *n* = 18). Substance-induced affective disorders were uncommon (2.9%, *n* = 25), and a small number of other disorders, including eating disorders, organic disorders and physiological effects of substance use, accounted for the remaining 2.5% (*n* = 14).

**Table 1:** Primary diagnoses and all diagnostic categories

Frequency of primary diagnoses				Frequency of all diagnosis			
Diagnostic group	*n* (%)	DSM diagnosis	*n* (%)	Diagnostic group	*n* (%)	DSM diagnosis	*n* (%)
No Axis I disorder	193 (34.6%)	Not applicable	Not applicable	No Axis I disorder	193 (26.5%)	Not applicable	Not applicable
Depressive disorders	127 (22.8%)	Major depressive disorder	108 (85.1%)	Substance use disorders	164 (22.5%)	Alcohol use disorder	75 (45.7%)
		Dysthymic disorder	12 (9.4%)			Cannabis use disorder	58 (35.4%)
		Recurrent depressive disorder	7 (5.5%)			Polysubstance use disorder	22 (13.4%)
Anxiety disorders	101 (18.1%)	Social phobia	29 (28.7%)			Cocaine use disorder	4 (2.4%)
		Obsessive–compulsive disorder	19 (18.8%)			Opiate use disorder	2 (1.2%)
		Generalised anxiety disorder	12 (11.9%)			Sedative use disorder	2 (1.2%)
		Panic disorder	11 (10.9%)			Stimulant use disorder	1 (0.6%)
		Post-traumatic stress disorder	8 (7.9%)	Anxiety disorders	162 (22.2%)	Social phobia	50 (30.9%)
		Body dysmorphic disorder	6 (5.9%)			Generalised anxiety disorder	28 (17.3%)
		Adjustment disorder	5 (5.0%)			Obsessive–compulsive disorder	23 (14.2%)
		Somatoform disorder	5 (5.0%)			Panic disorder	17 (10.5%)
		Anxiety disorder, not otherwise specified	5 (5.0%)			Post-traumatic stress disorder	12 (7.4%)
		Dissociative disorder	1 (1.0%)			Anxiety disorder, not otherwise specified	11 (6.8%)
Substance use disorders	82 (14.7%)	Alcohol use disorder	44 (53.7%)			Adjustment disorder	7 (4.3%)
		Cannabis use disorder	25 (30.5%)			Body dysmorphic disorder	6 (3.7%)
		Polysubstance use disorder	10 (12.2%)			Somatoform disorder	6 (3.7%)
		Cocaine use disorder	2 (2.4%)			Agoraphobia	1 (0.6%)
		Opiate use disorder	1 (1.2%)			Dissociative disorder	1 (0.6%)
Bipolar affective disorders	25 (4.5%)	Bipolar disorder, type 1	18 (72.0%)	Depressive disorders	149 (20.4%)	Major depressive disorder	126 (84.6%)
		Hypomanic episode	4 (16.0%)			Dysthymic disorder	13 (8.7%)
		Bipolar disorder, not otherwise specified	2 (8.0%)			Recurrent depressive disorder	10 (6.7%)
		Bipolar disorder, type 2	1 (4.0%)	Bipolar affective disorders	25 (3.4%)	Bipolar disorder, type 1	18 (72.0%)
Substance-induced affective disorders	16 (2.9%)	Substance-induced bipolar disorder	14 (87.5%)			Hypomanic episode	4 (16.0%)
		Substance-induced depressive disorder	2 (12.5%)			Bipolar disorder, not otherwise specified	2 (8.0%)
Other	14 (2.5%)	Anorexia nervosa	4 (28.6%)			Bipolar disorder, type 2	1 (4.0%)
		Bulimia nervosa	3 (21.4%)	Substance-induced affective disorders	19 (2.6%)	Substance-induced bipolar disorder	15 (78.9%)
		Delirium	2 (14.3%)			Substance-induced depressive disorder	2 (10.5%)
		Schizotypal personality disorder	2 (14.3%)			Substance-induced anxiety disorder	2 (10.5%)
		Acute intoxication	2 (14.3%)	Other	17 (2.3%)	Anorexia nervosa	7 (41.2%)
		Acute substance withdrawal	1 (7.1%)			Bulimia nervosa	3 (17.6%)
						Acute intoxication	2 (11.8%)
						Delirium	2 (11.8%)
						Schizotypal personality disorder	2 (11.8%)
						Acute substance withdrawal	1 (5.9%)

Multimorbidity was prevalent, with 36.4% of those ‘not meeting criteria’ with any Axis I diagnosis having a secondary Axis I diagnosis (*n* = 133), and a further 10.4% also having a tertiary diagnosis (*n* = 38). The cumulative frequency of all Axis I diagnoses met as either primary, secondary or tertiary diagnosis are displayed in Table 1. Substance use disorders were common as a secondary or tertiary diagnosis, and approximately a quarter of the sample (22.5%, *n* = 164) met criteria for a substance use disorder when all diagnoses were considered. Apart from substance use disorders, anxiety and depressive disorders continued to be the predominant illness categories when all diagnoses were considered (anxiety disorders: 22.2%, *n* = 162; depressive disorders: 20.4%, *n* = 149).

### Diagnostic change in the ‘non-case’ cohort

Of the ‘not meeting criteria’ cohort, 7.3% (*n* = 41) were referred for re-assessment on the basis that there was a new or evolving concern about their symptoms. At re-assessment, 44% (*n* = 18) met criteria for case status. Of the 19 individuals who continued to not meet criteria following re-assessment, four were re-referred for a third assessment. Three of these individuals were found to be experiencing a psychotic disorder. This resulted in 3.8% (*n* = 21) of the ‘not meeting criteria’ cohort who were known to develop psychosis and be referred to the service. The median time to first re-referral was 25 months (interquartile range (IQR): 33.5) and to second re-referral was 33 months (IQR: 50.25). Aggregated median duration from index to final reassessment was 27 months (IQR: 37).

### Demographic and socio-occupational attributes of the ‘not meeting criteria’ cohort

The demographic characteristics of both cohorts are listed in Table 2. Compared with the FEP group, the ‘not meeting criteria’ cohort was younger (mean age 29.8 *v*. 33.9 years), with a greater proportion of males (65.1% *v*. 57.4%) and those of Irish nationality (89.7% *v*. 82.5%). They had a broadly similar proportion of individuals with a family history of psychosis compared with the FEP group (12.0% *v*. 13.2%). There was a difference in the proportion of cases referred between secondary mental health services, with Dublin South East referring fewer than Dublin South (Cluain Mhuire) (odds ratio 0.64, 95% CI 0.49–0.84), which was broadly in line with the remaining services. Individuals ‘not meeting criteria’ were less likely to be either voluntary in-patients (odds ratio 0.31, 95% CI 0.24–0.39) or involuntary in-patients (odds ratio 0.10, 95% CI 0.06–0.15).

**Table 2:** Case and non-case characteristics, univariate and multivariate analysis

		Case (FEP)	Non-case	Univariate regression		Multivariate regression	
Variable	Categories	*n* (%)	*n* (%)	*P*-value	Crude odds ratio (95% CI)	*P*-value	Adjusted odds ratio (95% CI)
Demographic and treatment variables							
Gender	Female	525 (42.6)	195 (34.9)				
	Male	707 (57.4)	363 (65.1)	0.002	1.12 (1.03–1.71)		
Age	Mean (s.d.) years	33.9 (12.2)	29.8 (11.5)	<0.001	0.97 (0.96–0.98)		
Nationality	Irish	969 (82.5)	494 (89.7)				
	Non-Irish	206 (17.5)	57 (10.3)	<0.001	0.85 (0.77–0.91)		
Family history of psychosis	Yes	163 (13.2)	67 (12.0)				
	No	1070 (86.8)	491 (88.0)	0.493	1.08 (0.87–1.34)		
Catchment area	Dublin South (Cluain Mhuire)	465 (38.1)	230 (41.4)				
	Dublin South East MHS	340 (27.9)	108 (19.5)	0.001	0.64 (0.49–0.84)		
	Wicklow MHS	259 (21.2)	164 (29.5)	0.540	1.28 (0.99–1.65)		
	SJOG Independent	156 (12.8)	53 (9.5)	0.035	0.69 (0.48–0.97)		
Treatment status	Out-patient	463 (37.7)	403 (73.5)				
	Voluntary in-patient	443 (36.1)	118 (21.5)	<0.001	0.31 (0.24–0.39)		
	Involuntary in-patient	321(26.2)	27 (4.9)	<0.001	0.10 (0.06–0.15)		
Socio-functional variables							
Relationship status	In a relationship	271 (22.4)	94 (17.1)				
	Not in a relationship	940 (77.6)	455 (82.9)	0.012	1.10 (1.02–1.18)	0.913	1.02 (0.70–1.48)
Living status	Family/parents	576 (48.4)	316 (62.6)				
	Spouse/partner	157 (13.2)	58 (11.5)	0.267	0.72 (0.40–1.29)	0.632	0.86 (0.46–1.61)
	Other unrelated adults	200 (16.8)	39 (7.7)	0.835	1.07 (0.57–2.02)	0.736	0.88 (0.43–1.82)
	Alone	213 (17.9)	75 (14.9)	0.035	2.03 (1.05–3.92)	0.025	2.23 (1.10–4.48)
	Temporary or NFA	43 (3.6)	17 (3.4)	0.714	1.12 (0.60–2.08)	0.650	0.86 (0.44–1.66)
Employment or education status	Working or education	505 (42.3)	265 (49.3)				
	Not working (≤12 months)	449 (37.6)	119 (22.1)	0.753	1.08 (0.67–1.73)	0.239	1.38 (0.81–2.340
	Not working (>12 months)	188 (15.7)	124 (23.1)	0.002	2.14 (1.31–3.50)	<0.001	2.83 (1.65–4.85)
	Unable to work	53 (4.4)	30 (5.6)	0.550	0.86 (0.52–1.42)	0.712	1.11 (0.64–1.90)

FEP, first-episode psychosis; MHS, mental health service; SJOG, St. John of God; NFA, no fixed abode.

Social and occupational functioning was examined across three variables. The ‘not meeting criteria’ group were more likely not to be in a relationship, but not at a level of significance when adjusted for demographic factors (adjusted odds ratio 1.02, 95% CI 0.70–1.48). They had a similar distribution of living arrangements, with the majority of both cohorts continuing to live with their families of origin. A substantial proportion of both cohorts were not working at the time of assessment, with a significant finding of a higher proportion of long-term unemployment (≤12 months) in the ‘not meeting criteria’ cohort, which endured in the adjusted model (adjusted odds ratio 2.83, 95% CI 2.83–4.85).

## Discussion

### Main findings

This paper is one of the few to report on the cohort of individuals referred to an EIP service, but not meeting criteria for either an ARMS or FEP. Our results demonstrate that this group constitute a large proportion of the total referrals to an EIP service, substantially larger than those meeting ARMS criteria.

The ‘not meeting criteria’ cohort was highly enriched for non-psychotic Axis I disorders, with approximately 65% meeting criteria for a least one disorder, with mood, anxiety and substance use disorders predominating. This finding is consistent with a previous study in this service over a more limited timeframe.^28^ A further third (36.4%) had at least a secondary diagnosis. A population-based study exploring longitudinal risk demonstrated that prior psychopathology accounted for 85.5% of the outcome of developing clinical psychosis. Mood disorders have the highest population attributable fraction, which was the most common diagnostic group in our cohort. Although the relative risk for psychosis with an ARMS is high, because of its lower prevalence in the general population compared with more common mood, anxiety and substance use disorders, its population attributable fraction is far lower.^20^ Non-psychotic pathology has also been reported as high in ARMS groups,^29,30^ which may contribute substantially to the conversion risk in this group.^31^ Our findings demonstrate that highly enriched Axis I morbidity and multimorbidity is a finding that is not specific to ARMS groups among EIP referrals.

The socio-occupational functioning of the ‘not meeting criteria’ cohort was examined across three variables. Although a greater proportion were not in a relationship, this association dissipated when controlled for demographic factors, likely reflecting their younger age. They were broadly similar to the FEP cohort across other metrics, with the exception of a greater proportion in long-term unemployment (adjusted odds ratio 2.83, 95% CI 1.65–4.85). The broad similarity to an FEP cohort, a group with established socio-occupational impairments,^32^ is a reflection of the functional morbidity in this group. Impaired social and role functioning have predictive capacity for psychosis, both within ARMS^33^ and general population^23^ samples. The significance of functional impairment as a risk marker is not only its capacity to define at-risk individuals, but also its presence as the earliest herald symptoms in the pre-illness phase of individuals with psychosis.^34^ Risk stratification at this point offers the potential for intervention at the earliest possible stage.

Despite the fact that our FEP cohort had a majority of males, our ‘not meeting criteria’ cohort had a greater proportion again (odds ratio 1.12, 95% CI 1.03–1.71). Although classically, schizophrenia-like psychosis was thought to have a higher incidence in men, this relationship moderates when incidence is considered over the lifespan and psychotic syndromes considered are broadened. However, an age×gender interaction has been demonstrated, reflecting that before 45 years of age, overall risk rates are elevated among men.^35^ This renders our finding of the ‘not meeting criteria’ group as predominately both male and younger as significant. Clinical and functional outcomes in psychosis are also worse in men.^36^

Ethnicity is a well-established risk factor for psychosis, with differential risk between groups.^35^ Our catchment area has previously been reported as having a marginally higher than average migrant population,^37^ in line with that reported for this FEP cohort. When all ‘non-Irish’ nationalities were collapsed into a single category, the ‘not meeting criteria’ group had a lower proportion of non-Irish nationalities (odds ratio 0.85, 95% CI 0.77–0.91), indicating that this variable is unlikely to be a source of elevated risk. A family history of a psychotic disorder is a further well-established risk factor.^38^ There was a similar proportion of both cohorts with a first- or second-degree relative with a psychotic disorder, indicating that this risk factor is equivalent between cohorts.

Finally, our study found that of those initially not meeting criteria, 41 were re-referred to the service, with 21 subsequently meeting criteria for psychosis (3.8% of all those ‘not meeting criteria’). This figure should be interpreted with caution and it is not synonymous with a conversion to psychosis rate in the sample. It is likely to be an underestimate of the true conversion rate of this cohort. Reasons for non-detection by our service of individuals who developed psychosis subsequent to assessment include being lost to care (e.g. disengaging with services or moving out of the catchment area) or treating secondary care teams not re-referring to the service (e.g. if an individual was re-referred by primary care, but the secondary care team deemed them to no longer be in their FEP). The variable duration from assessment to the end of the study period may also have been an inadequate interval for recently enrolled individuals to develop psychosis. One of the few studies to report on conversion to psychosis rates among an entire cohort of EIP service presenters found that over a median follow-up period of 7.3 years, the conversion to psychosis rate was comparable between ARMS (17.3%) and non-psychotic help-seekers (14.6%). Non-psychotic help-seekers with a previous admission had a higher rates of psychosis post-assessment than the ARMS group.^39^ The reported service differed from ours in several important ways, accepting referrals for adolescents aged 12–25 years and employing a pre-screening interview. To establish a definitive figure for psychosis risk in our cohort, a retrospective cohort study with longitudinal follow-up should be undertaken.

Our age criteria of 18–65 years mirrors the configuration of secondary mental health services in Ireland, and is aligned with adult service age criteria. This differs from some EIP services that focus on an adolescent and young adult population, often in the 14- to 35-year age range. In the context of the lower prevalence of psychotic disorders in the <18 year age group, the issues we raise previously, of both the limited predictive ability and capacity of current ARMS models, have been highlighted as particularly relevant in this group.^40^ As we anticipate that those ‘not meeting criteria’ would constitute a larger proportion of referrals within adolescent EIP service referrals, it would be instructive to repeat our study methodology in such a service.

EIP services have several functions distinct from identifying psychosis risk, including delivering phase-specific, specialist interventions for at-threshold psychotic disorders. In this regard, the high volume of individuals identified by clinical teams as experiencing an FEP and appropriately referred for EIP interventions, remains an important and essential contribution of the service.

In a similar vein, where risk prediction is implemented, it should be for the purpose of delivering evidence-based, risk-modifying interventions. The development of risk prediction models is driven by broad acknowledgement that psychosis has developmental and symptomatic antecedents,^41^ and that intervening early may engender the best clinical outcomes. Such enquires are catalysed by outcomes for psychotic disorders, which have largely plateaued in recent decades.^42^ There is emerging evidence that screening in children and adolescents, when based on a combination of symptomatic and functional markers (distinct from either in isolation), has acceptable predictive power for later psychopathology, and that there are some promising risk-modifying interventions within this interval.^43^ In the adult population, the evidence is less clear. Currently there are no definitive data supporting any risk-modifying intervention in adults,^44^ nor do antipsychotics appear to have a role in modifying transition rates in ARMS samples.^45^ Such studies may be challenged by the same issues discussed previously of high heterogenicity within high-risk samples, uncontrolled risk enrichment and uncertain representativeness of current ARMS cohorts for incident FEP cases.

At such a juncture, clinical concerns about prediction beget ethical ones. Models that induct individuals into a surveillance pathway, where a majority (as currently) will be ‘false positives’, raise important issues about stigmatisation and iatrogenic harm.^5^ Risk prediction models are currently not accurate enough for clinical use, and the risk of adverse effects for those who screen false positive should not be underestimated. These questions are not peripheral to, but inextricably linked with, the performance of such models, as there are careful balances to be made between potential benefit and harm. Continuing research efforts need to address each aspect of risk prediction not in isolation, but in tandem, including discriminative ability, calibration and, ultimately, clinical utility.^12^

### Study strengths

This study includes complete referral data from a long-established EIP service, embedded in a public mental health service that provides universal access to the catchment area population. This allowed the examination of case trends over a broad timeframe, resulting in a large sample size.

Our service provides a standardised diagnostic assessment, using a recognised instrument (SCID), to all individuals referred. This allows for the reporting of those meeting standardised criteria for many psychotic and non-psychotic disorders. Standardised assessment templates with data on functional domains facilitated a transdiagnostic comparison between cohorts.

Finally, since the inception of the service, an electronic archive of assessment documents has been maintained, resulting in a high data retrieval rate.

### Study limitations

As EIP services vary in case criteria and service configuration, caution should be exercised in generalising findings across health service models. Our service functions as a tertiary referral service and does not accept direct referrals from primary care or self-referrals. In services where this is the case, it might be expected to increase the proportion of those ‘not meeting criteria’, with a likely dilutional effect on risk factor enrichment.

Although our study provided a comprehensive description of DSM Axis I disorders, we do not assess for other conditions with standardised assessment instruments, including Axis II disorders (including personality disorders) or neurodevelopmental disorders (including attention-deficit hyperactivity disorder or autism spectrum disorder). It is possible that some of the individuals who did not meet criteria for an Axis I disorder would meet criteria for one of these disorders, and for those who did meet criteria for a disorder, it may further describe their comorbidities.

Finally, as above, an accurate figure for conversion to psychosis should be established for this group, with longitudinal follow-up. Although a portion of those developing psychosis will have been re-referred, several factors affecting service engagement limit the utility of this figure.

### Implications and future research

In conclusion, to our knowledge, this is largest study to report on demographic, diagnostic and functional variables of individuals referred to, but not meeting criteria for, an EIP service. We demonstrated that this cohort is enriched for many risk factors for psychosis. Enhanced models of psychosis risk derived from current ARMS criteria may succeed in enhancing predictive capacity within selected groups, but will not address the ‘prevention paradox’ of only a limited portion of individuals entering psychosis via this route. This study is a response to calls to reconsider the current ARMS model as a starting point for defining at-risk groups, to build models that will be useful at the population level. Those referred to EIP service, but who do not meet current criteria, may be an important group for further study in this expanded search.

## Data Availability

The data that support the findings of this study are available from the corresponding author, S.N., upon reasonable request.
